# Does angiotensin-1 converting enzyme genotype influence motor or cognitive development after pre-term birth?

**DOI:** 10.1186/1742-2094-2-6

**Published:** 2005-02-22

**Authors:** David R Harding, Sukhbir Dhamrait, David Devadason, Steve E Humphries, Andrew Whitelaw, Neil Marlow, Hugh E Montgomery

**Affiliations:** 1 Neonatal Intensive Care Unit, St. Michael's Hospital, Bristol, UK; 2 Division of Cardiovascular Genetics, University College London, London, UK; 3 University of Bristol Medical School, Southmead Hospital, Bristol, UK; 4 School of Human Development, University of Nottingham, Nottingham, UK

## Abstract

**Background:**

Raised activity of the renin-angiotensin system (RAS) may both amplify inflammatory and free radical responses and decrease tissue metabolic efficiency and thus enhance cerebral injury in the preterm infant. The angiotensin-converting enzyme (ACE) DD genotype is associated with raised ACE and RAS activity as well as potentially adverse stimuli such as inflammation. The DD genotype has been associated with neurological impairments in the elderly, and thus may be also associated with poorer motor or cognitive development amongst children born preterm prematurely.

**Methods:**

The association of DD genotype with developmental progress amongst 176 Caucasian children born at less than 33 weeks gestation (median birthweight 1475 g, range 645–2480 g; gestation 30 weeks, range 22–32; 108 male) was examined at 2 and 5 1/2 years of age. Measured neuro-cognitive outcomes were cranial ultrasound abnormalities, cerebral palsy, disability, Griffiths Developmental Quotient [DQ] at 2 yrs, and General Cognitive Ability [British Ability Scales-11] and motor performance [ABC Movement], both performed at 5 1/2 yrs. All outcomes were correlated with ACE genotype.

**Results:**

The DD genotype was not associated with lower developmental quotients even after accounting for important social variables.

**Conclusion:**

These data do not support either a role for ACE in the development of cognitive or motor function in surviving infants born preterm or inhibition of ACE as a neuroprotective therapy.

## Background

Delight over recent survival gains for the very premature infant has been tempered by the frequent presence of cerebral injury and developmental impairment. One quarter of those born before 26 weeks postmenstrual age (at least 11 weeks premature) show evidence of severe cerebral injury including cognitive dysfunction by 30 months of age [1]. Preterm children without any disability remain at risk of a range of motor, cognitive, behavioural and psychological deficits during childhood even if not born so close to the margin of viability [2]. To date, the pathophysiological processes leading to such impairment remain largely occult. In particular, cerebral imaging has failed to identify structural correlates of impaired higher function [3] although imaging can predict many cases of motor abnormality (such as cerebral palsy) due to the presence of periventricular white matter injury [4].

Three factors seem to play important roles in the aetiology of preterm cerebral injury. Firstly, exposure to inflammatory stimuli is associated with white matter injury and cerebral palsy in the preterm [5]. Secondly, reduced glucose and oxygen delivery to the developing brain (hypoxia-ischaemia: local cerebral or systemic) may cause excito-toxic neurotransmitter release followed by neuronal death [6]. Thirdly, free-radicals may damage the oligodendrocytes of white matter of the preterm brain [6]. Damage to the primitive white matter prevents the normal formation of grey matter connections which may influence cognitive development in childhood [7].

Candidate systems that might influence motor or cognitive outcome after premature birth are likely to be those which affect these responses. The human renin-angiotensin systems may be such a system. Angiotensin converting enzyme (ACE), a key component of the circulating (or endocrine) renin-angiotensin system (RAS), cleaves angiotensin I to yield the potent vasoconstrictor angiotensin II. In addition, ACE degrades vasodilator kinins. In these ways, endocrine RAS plays an important role in circulatory homeostasis. However, local RAS also exist in diverse human tissues including lung, myocardium, vasculature, lymphocyte and brain tissue. These are powerful regulators of mitochondrial respiration and whole-cell metabolism [8] and exert profound effects on whole-human metabolism and metabolic efficiency: elevated ACE may impair cellular aerobic metabolism [9]. RAS also plays a key role in the regulation of tissue inflammatory responses; ACE, through generation of angiotensin II, stimulates the synthesis of pro-inflammatory cytokines, including IL-6 which itself is thought to exert major neurocytotoxic effects with the genesis of functionally significant lesions in the developing preterm brain [5]. It has also been noted that the inhibition of RAS may reduce the effects of excitotoxic neurotransmitters and free radicals [10]. It is possible therefore that enhanced ACE activity may adversely influence the development of the child born prematurely.

A common variant of the human ACE gene provides a tool to determine if ACE activity does influence developmental progress after preterm birth. The presence (insertion, or 'I' allele) rather than the absence (deletion, or 'D' allele) of a 284-base-pair fragment in the human ACE gene is associated with lower ACE activity in organs including both circulating inflammatory cells [11] and the circulation itself [12]. Given the likely causal association of pro-inflammatory responses, ischaemic-hypoxia, excitotoxic neurotransmitters, and free radical attack with impaired neuro-outcome; and given the potential role of increased RAS activity in amplifying these effects, we might expect the DD genotype (encoding raised ACE activity) to be associated with poorer neuro-developmental progress after pretem birth. Comparable findings have been described with respect to the deterioration of cognitive function in the elderly by some authors [13-15]. We have tested this hypothesis by studying the association of the ACE I/D polymorphism with measures of neuro-developmental progress at 2 and 5 1/2 years of age in children who had participated in a neuro-developmental outcome study (The Avon Premature Infant Project, APIP [16]). All the patients were born at less than 33 weeks postmenstrual age (normal gestation is 37–40 weeks).

## Methods

### Patients

The study was approved by the ethical committees of Southmead Hospital and United Bristol Health Care Trust. Parental consent was obtained for participation in neurodevelopmental follow-up [16] (see below). Consent was not required for the genetic component of this study as all personal information was held separately from the genetic information and patients were identified only by study codes.

All children were born at 32 weeks gestation or less, between December 1990 and July 1993 at Southmead Hospital or St. Michael's Hospital, Bristol. All had participated in the Avon Premature Infant Project (APIP) [16]. Briefly, this was a randomised controlled trial in which developmental support (Portage) or supportive counselling (parental adviser), each started at discharge and continued for up to 2 years, were found to confer some measurable (3–4 DQ points (below)) but clinically insignificant benefit to development at 2 years of age, when given in addition to appropriate primary care and community support, after adjusting for social variables.

### Neuro-developmental outcome

The Griffiths Mental Development Scales, used to assess motor and cognitive performance, was performed at 2 years corrected age [17]. The Griffiths scales comprise five subscales, including personal and social, hearing and speech, locomotor, eye hand co-ordination and performance domains, from which is derived an overall developmental quotient (DQ). A lower Griffiths DQ reflects a poorer neuro-developmental performance, with a difference DQ of five points being clinically apparent. DQ was standardised originally to a mean of 100, with a standard deviation of 15, but secular drifts in population scores have resulted in a higher population mean. Thus for severe disability a score of 70 (-2 standard deviations (sd)) would indicate severe disability. Cognitive developmental progress at 5.5 years of age was assessed using the British Ability Scales [18]. The BAS-II was standardised in the early 1990s and was used to compute general cognitive ability (GCA) together with visuospatial, verbal and non-verbal subscales. The GCA is a developmental quotient, equivalent to an IQ estimate, normalised at 100 (sd +/- 15) in which a lower score again indicates poorer conceptual ability. The Movement ABC scales were used to assess manual dexterity, ball skills, and balance over ten tests at 5 1/2 years of age. Scores of each component are summed to produce a composite score ranging from 0–40, with high scores indicating a more impaired motor skills and 0 indicating normal skills.

A psychologist performed the Griffiths Scales of Mental Development and a second psychologist performed the British Ability Scales (second edition) (BAS). The ABC Movement tests were performed by a trained research nurse. All assessments were blind to the child's neonatal course and subsequent progress.

### ACE genotyping

DNA was extracted from the Guthrie card blood spots (newborn metabolic screening cards). ACE genotype was determined using 3-primer PCR amplification [9]. Primer ratios corresponded to 50 pmol of an I-specific oligonucleotide in a 20-υl reaction volume. The PCR was performed using Taq polymeraase yielding amplification products of 84 bp for the D allele, and 65 bp for the I allele. Amplification products were visualised using a 7.5% polyacrylamide gel stained with ethidium bromide. Genotyping was performed by staff blind to all clinical data.

### Study Size

An estimate of sample size suggested that 144 patients would be needed for this study. The assumptions made for this calculation were that DD genotype infants had a mean DQ of 92.5 (1/2 SD below the norm) compared to a mean DQ of 100 in the ID+II group, assumed typical genotype distributions, and a significance of 0.05 with 80% power.

### Statistical analysis

Data were stored in SPPS v9.0 for Windows. Lymphocyte [11] and tissue ACE [12] activity is primarily raised in DD genotype when compared to either ID or II genotype, and so data for those of DD genotype were compared to those from I-allele carriers. Categorical data were analysed by Chi square and continuous data by Student's T Test if normally distributed or Mann-Whitney U test as appropriate.

## Results

Guthrie cards were located for 230 of 308 children. After exclusion of non-Caucasians and, at random, 1 child of any identical twin pairs (based on genotypes and gender) 176 babies with ACE genotype formed the study population (median birthweight 1475 g, range 645–2480 g; gestation 30 weeks, range 22–32) with follow-up data at 2 years. 122 of these also had follow-up at 5 1/2 years. The ACE genotype distribution was 49 [27.8%] DD, 73 [41.5%] ID, 54 [30.7%] II, demonstrated Hardy-Weinberg equilibrium, and was similar to that observed in the newborn term population from the same region of the UK (203 [24.1%] DD, 433 [51.5%] ID, 205 [24.4%) II). Baseline characteristics were independent of genotype, except that fewer individuals of DD genotype were from twin births (*p *= 0.047) (table 1). There was no association between markers of neonatal cerebral injury: severe intraventricular haemorrrhage or white matter injury (table 1). There was no association with the presence of any disability at 2 years of age (DD 17% vs ID/II 15%, *p *= 0.65).

**Table 1:** Perinatal and social factors

	DD Genotype (n = 49)	ID/II Genotype (n = 127)
		
No maternal antenatal corticosteroids	44 (80%)	112 (81%)
No. of children from twin pregnancy*	4 (8%)	27 (21%)
Male	32 (65%)	76 (60%)
Gestation, weeks (± SEM)	29.7 (± 0.3)	30.0 (± 0.2)
Birth weight, g (± SEM)	1453 (± 56)	1461 (± 34)
Portage, parent adviser	17 (31%), 19 (35%)	46 (33%), 42 (30%)
Severe intraventricular haemorrhage	5 (11%)	7 (6%)
White matter injury	7 (14%)	14 (11%)
Maternal age (± SEM)	27.2 (± 0.8)	27.4 (± 0.8)
Manual occupation	28 (57%)	76 (60%)
Maternal car use	30 (61%)	75 (60%)
Mother educated beyond 16 yrs.	17 (35%)	48 (38%)

*p = 0.047 (Fisher's Exact Probability Test)

Continuous data is shown as mean (± standard error of mean).

Measures of developmental cognitive and motor outcome were entirely independent of genotype (table 2). The findings were unchanged after post hoc subgroup analysis of singletons, infants with normal cranial scans, amongst children without disability and after adjusting for potential influential variables (including twin birth) using multiple regression (data not shown).

**Table 2:** ACE genotype and developmental performance at 2 and 5 1/2 years of age. Data shown is mean (± SEM).

Developmental tests	DQ for DD	DQ for ID/II	*p*
			
Griffith DQ at 2 years	96.2 (3.1)	96.3 (1.3)	0.95
			
Locomotor subscale	92.7 (2.7)	92.4 (1.3)	0.92
Personal & social subscale	101.9 (3.0)	101.0 (1.6)	0.80
Hearing and speech subscale	92.9 (4.1)	94.0 (2.1)	0.80
Eye hand co-ordination subscale	90.8 (3.1)	92.8 (1.2)	0.46
Performance subscale	102.2 (4.3)	101.3 (1.6)	0.79
			
Griffith DQ at 2 years (adjusted for social variables)	100.0 (0.9)	99.3 (0.6)	0.43
			
ABC Movement summative score	8.1 (1.8)	8.0 (0.9)	0.97
			
GCA at 5 1/2 years	99.2 (3.4)	100.2 (2.0)	0.80
			
Verbal ability subscale	98.0 (4.0)	103.2 (1.7)	0.22
Pictoral ability subscale	99.9 (3.3)	98.7 (1.7)	0.99
Spatial ability subscale	98.4 (3.3)	97.3 (1.9)	0.67

## Discussion

After a search of Embase and Medline we believe that this study is the first to attempt to dissect out the contribution of genetic variation in the ACE gene to developmental progress after pre-term delivery. Despite much physiological and biochemical evidence to support our hypothesis, we found that ACE DD genotype was not associated with adverse long term developmental outcome in infants of < 33 weeks gestation in this study.

These data are perhaps at variance with previous studies of Alzheimer's disease, age-associated memory impairment and vascular dementia, all of which have implicated the ACE D allele in having a role in mental decline [13-15]. However this is not a universal finding. Furthermore although ACE inhibitors appear to reduce inflammatory responses, ischaemic effects, and excitotoxic and free radical induced injury [10], angiotensin II does not (indeed angiotensin II may actually enhance ischaemic and excitotoxic neural injury via the AT2 receptor). In addition, both captopril and losartan (RAS inhibitors) appear to improve cognitive performance in mice [19] and humans [20]. It should be noted however that little is known about the ontogeny of the RAS in the human foetus. Certainly RAS (and angiotensin II receptors in particular) play a role in blood-brain barrier and central nervous system development in mice, and alterations in RAS receptor expression over foetal and neonatal life are recognised. It is thus possible that developmentally regulated patterns of AT1 receptor expression might offer some level of protection against the potentially detrimental effects of ACE-mediated angiotensin II synthesis.

Although there may be similar molecular pathways that effect cerebral injury in the preterm infant and the elderly, ontological differences in the expression of genes involved in predisposition to neural injury are well described. In particular reactive production of nitric oxide may be enhanced in the elderly and the ability to protect the brain from oxidants may be reduced in the elderly (22). Thus the effect of any one polymorphism, with a relatively minor effect, may be swamped in the newborn infant by other protective mechanisms.

The lack of any association between ACE genotype and scores of developmental progress was also surprising because we have demonstrated an association between DD genotype and markers of poor cardio-respiratory instability in the perinatal period in this patient group [21]. This association (between genotype and worse early cardio-respiratory status) could predispose to death, which would in turn weaken any association (if it exists) between DD genotype and worse developmental quotients. It is of course possible that our sample size was insufficient to demonstrate any association with ACE genotype and developmental progress. However, similar-sized studies have been sufficient to demonstrate an association between ACE D allele and cognitive decline in the elderly [13-15], and power calculations suggested we had enough patients to demonstrate at least a trend. If an undetected genotype-association does exist such an effect is weak.

## Conclusion

We cannot support an association of ACE genotype with cognitive or motor development in survivors born preterm or, thus, the use of RAS inhibition as a neuroprotective agent in the preterm. Given the current lack of understanding of the mechanisms leading to cerebral injury and subsequent impairment – particularly of higher function – in such patients, further genetic association studies of other candidate genes are warranted.

## List of abbreviations used

ACE, angiotensin-1 converting enzyme; DQ developmental quotient, BAS, British ability scales (second edition); GCA, general cognitive ability; RAS, renin angiotensin system; PCR, polymerase chain rection.

## Competing interests

The author(s) declare that they have no competing interests.

## Authors' contributions

DH, HM, AW, NM conceived the study and its design and wrote the manuscript. DH, DD and SD performed data collection, DNA extraction and PCR and participated in analysis of the data with SH and HM. NM reviewed all cranial imaging. All authors participated in the writing of the manuscript and approved the final manuscript.
